# Biological mechanisms governing the periodontal regenerative microenvironment: cellular crosstalk, extracellular matrix remodelling, and immunomodulation

**DOI:** 10.3389/fcell.2026.1891657

**Published:** 2026-07-14

**Authors:** Qi Cui, Fengxiang Li, Xia Zhao

**Affiliations:** Qingdao Stomatological Hospital Affiliated to Qingdao University, Qingdao, Shandong Province, China

**Keywords:** cementum-PDL-bone complex, extracellular matrix remodelling, immunomodulation, macrophage reprogramming, periodontal ligament stem cells, periodontal regeneration, regenerative microenvironment, responsive biomaterials

## Abstract

Periodontal regeneration is no longer viewed as the simple replacement of lost alveolar bone, but as the reprogramming of a diseased regenerative niche. Predictable repair requires coordinated reconstruction of cementum, periodontal ligament (PDL), and alveolar bone within a chronically infected, inflamed, and mechanically loaded microenvironment. Unlike regeneration in bone, skin or muscle, periodontal regeneration requires reconstruction of an integrated cementum-PDL-bone unit. Success therefore depends not on repair of a single tissue compartment, but on coordinated cementogenesis, PDL fibre insertion, alveolar bone remodelling, vascularization, immune resolution and restoration of load-bearing tooth support. Here, we synthesize current evidence on the cellular, matrix-based, and biomaterial mechanisms that regulate this process. Periodontal ligament stem cells, PDL fibroblasts, macrophages, endothelial cells, osteoclast-lineage cells, and resident progenitors form interconnected signalling networks mediated by cytokines, chemokines, extracellular vesicles, apoptotic bodies, mitochondrial signals, metabolites, and extracellular matrix cues. In periodontitis, these networks are disrupted by microbial dysbiosis, persistent inflammation, oxidative stress, stromal senescence, metabolic dysfunction, osteoclastogenesis, and collagen degradation, collectively limiting osteogenesis, cementogenesis, angiogenesis, and PDL fibre organization. Effective regeneration should therefore proceed through staged control of infection, resolution of inflammation without loss of host defence, restoration of stem-cell fitness, recruitment of vascular and mesenchymal progenitors, and matrix remodelling toward functional tissue integration. Responsive biomaterials, including hydrogels, metal-organic frameworks, nanozymes, vesicle-based platforms, and bioelectric or piezoelectric matrices, may help couple local pathological cues to controlled therapeutic release. Clinical translation will require standardized disease models, spatial and single-cell biomarkers, mechanism-defined potency assays, and endpoints that measure cementum-PDL-bone integration rather than bone fill alone.

## 1 Introduction

The periodontium should be thought of as an organ rather than a series of tissue compartments. Its function relies on the coordinated interaction of alveolar bone, cementum, periodontal ligament (PDL), gingival connective tissue, vascular and neural networks and an immune system continuously challenged by the oral micro biome. This coordination gradually breaks down during periodontitis. Chronic inflammation, osteoclast mediated bone loss, oxidative injury, extracellular matrix ECM degradation and depletion of local pool of regenerative cells through microbial dysbiosis occurs. In this perspective no one can talk about restoring the bone defects as defect filling; they cannot even talk about the periodontal regeneration itself as defect filling. It involves reconstruction of a mechanically integrated and immunologically stable cementum-PDL-bone complex.

This distinction is central to the present review. Bone regeneration is often assessed by mineralized tissue volume and mechanical continuity, skin repair by epithelial closure and dermal matrix restoration, and muscle regeneration by myofibre renewal and neuromuscular function. Periodontal regeneration has a different requirement, as it must rebuild an interfacial organ in which mineralized cementum and alveolar bone are connected by obliquely oriented, vascularized and innervated PDL fibres. This regenerated unit must also withstand oral microbial challenge and continuous occlusal loading. We therefore interpret cellular crosstalk, biomaterial design, immunomodulation and metabolic regulation according to whether they restore the integrated cementum-PDL-bone complex, rather than whether they merely improve bone fill or generic tissue repair.

This concept has reshaped the direction of regenerative research. These previous treatments were primarily barrier membranes, grafts, enamel matrix derivatives or transplanted cells. The newer strategies attempt to rechannel altered lesions towards repair in the developmental process. This paradigm shift has been termed as from reparative manipulation to developmental engineering of periodontal regeneration (Liu G. et al., 2025). The single cell data also reveals the presence of heterogeneous stromal, immune, endothelial and osteogenic populations in the periodontal lesions (Chen et al., 2022) including progenitor populations associated with angiogenesis and regeneration (Liu J. et al., 2025).

The evidence so far indicates three interconnected regulatory aspects. One involves ‘crosstalk’ between cells. While the periodontal ligament stem cells (PDLSCs) and PDL fibroblasts are involved in osteogenic, cementogenic, fibroblastic and immunmodulatory activities (Yang Y. et al., 2024; Zhao et al., 2022) their capability is affected by inflammatory diseases, diabetes and senescence (Xiao et al., 2026; Zhai et al., 2024; Jin et al., 2022; Queiroz et al., 2021). PDL fibroblasts can also display inflammatory characteristics which continue to cause damage to tissue (Huang et al., 2024). Macrophages also play a key role: they contribute to the production of different cytokines, osteoclastogenesis, oxidative stress and the behaviour of stromal cells (Yamaguchi, 2026; Peng et al., 2024; Wang C. et al., 2023; González-Quintanilla et al., 2026).

Another dimension is ECM remodelling. The ECM not only provides structural support but also conveys biochemical and mechanical information. The organization of collagen, its stiffness, mineral makeup, degradability, adhesive ligands and ion release can be used to alter stem-cell and immune-cell behaviour. Recently, many hydrogels and scaffolds have been incorporated with modes of ion or small molecule delivery, or antioxidants and nanozymes to respond to periodontal inflammation (Lin et al., 2025; Yang S. et al., 2024; Yu et al., 2026; Wang et al., 2025; Xu Y. et al., 2023). Other platforms add other bioactive ingredients such as exosomes, growth factors, gases or bioelectrical cues to help in vascular, osteogenic or immune repair (Santos et al., 2023; Liu X. et al., 2024; Fang et al., 2024; Liu et al., 2022).

Another level of regulation is the immune status of the defect site. Activated macrophages, T and B cells, receptor activator of nuclear factor-kappa B ligand (RANKL) mediated osteoclast formation, reactive oxygen species (ROS) and persistent production of cytokines characterize periodontal lesions. Repair is better when macrophages are polarized to reparative phenotypes or when activation of nuclear factor-kappa B (NF-kappaB) signalling is inhibited (Yamaguchi, 2026; Wang C. et al., 2023; Shi et al., 2026). Related strategies act through activation of nuclear factor erythroid 2-related factor 2 (NRF2), suppression of osteoclast activity or delivery of pro-resolving vesicles, each of which can reduce inflammation-associated bone loss (Wang et al., 2025; Xu Y. et al., 2023; Cui et al., 2023; Nakao et al., 2021). The goal is therefore not broad immunosuppression, but restoration of an immune response capable of resolving inflammation in a manner appropriate to the local tissue context and disease stage (Yang et al., 2021).

**FIGURE 1:**
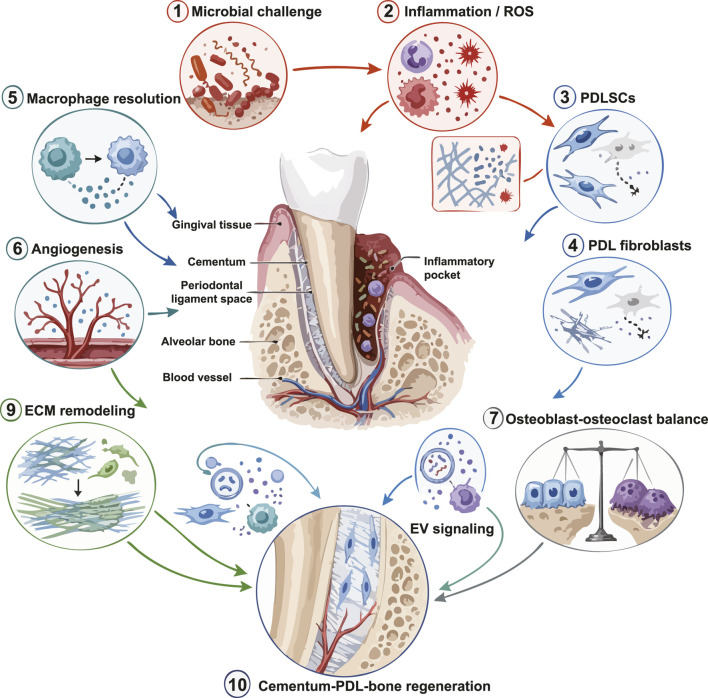
Cellular and extracellular matrix framework of the periodontal regenerative microenvironment.

Here, we frame periodontal regeneration as microenvironmental reprogramming. The translational question is which biological state a lesion must reach before the cementum-PDL-bone unit can regenerate predictably. To make this framework explicit, Table 1 maps the major cellular modules to the pathological barriers they encounter and the design priorities they imply. Figure 1 follows the same logic, tracing the sequence from microbial challenge and inflammatory injury to macrophage-mediated resolution, angiogenesis, ECM remodelling and reconstruction of the cementum-PDL-bone complex.

**TABLE 1:** Cellular logic of the periodontal regenerative microenvironment.

Cellular module	Regenerative contribution	Disease-related barrier	Design/Readout priority
PDLSCs	Osteogenic/cementogenic potential; local progenitor pool	Inflammation, diabetes, mitochondrial stress and senescence	Restore viability, mitophagy and lineage commitment
PDL fibroblasts	Collagen organization; mechanical and immune sensing	Inflammatory fibroblast states and matrix-degrading signals	Track fibroblast states, collagen alignment and PDL width
Macrophages	Microbial clearance; resolution signals; progenitor instruction	Persistent pro-inflammatory activation; simplified M1/M2 endpoints	Time immune switching and verify functional repair states
T/B-cell-RANKL axis	Controls osteoclastogenesis and bone turnover	Excess RANKL, cytokines and immune-driven resorption	Report RANKL/OPG balance and osteoclast activity
Endothelial cells	Angiogenic front; oxygen, nutrients and immune trafficking	Hypoxia, poor vascular ingrowth and immature vessels	Couple angiogenesis with stromal and matrix maturation
Osteoblast/osteoclast lineage	Bone formation and remodelling of mineralized tissue	Uncoupled resorption or bone fill without attachment	Assess bone quality together with cementum and fibre insertion
EVs/apoptotic bodies	Cell-free paracrine cargo; immune and metabolic signalling	Disease-derived vesicles may transfer harmful cargo	Define source, priming, cargo potency and safety markers

## 2 Cellular crosstalk in the periodontal regenerative niche

### 2.1 PDLSCs and PDL fibroblasts as gatekeepers of repair

PDLSCs are central to periodontal regeneration because they reside within the PDL niche and can generate osteogenic, cementogenic and ligament-associated lineages. Their self-renewal, multipotency and immunomodulatory activity have made them useful candidates for tissue engineering, cell-free therapies and other reparative strategies (Zhao et al., 2022; Queiroz et al., 2021). Work in neural-injury models also suggests that their plasticity extends beyond periodontal tissues (Mohebichamkhorami et al., 2022). Nonetheless, PDLSCs are not a homogeneous regenerative pool. Single-cell profiling has revealed diverse mesenchymal dental subsets, and variable cell-cycle states and lineage biases which vary with tissue context (Yang Y. et al., 2024). These cells are further influenced by inflammatory signals, microbial factors, and mechanical stress and metabolism in diseased periodontal deficiencies.

Fibroblasts of PDL also play an active role in the repair environment. They contribute to the maintenance of periodontal homeostasis and the local immune balance in physiological conditions. During disease, however, the same stromal compartment can release cytokines and contribute to tissue damage (Huang et al., 2024). Single-cell analyses of periodontitis have identified inflammatory fibroblast states, including *TNFRSF21*-positive cells enriched in CXCL-family chemokines and *IL24* (Chen et al., 2022). This suggests that stromal cells may help sustain the inflammatory circuit rather than simply respond to it. Osteogenic induction alone may therefore be insufficient if fibroblasts remain chemokine-rich, senescent or biased towards pro-osteoclastogenic signalling.

Inflammation also weakens the osteogenic capacity of PDLSCs. Lipopolysaccharide and inflammatory cytokines interfere with osteogenic differentiation through several converging pathways (Jin et al., 2022). Some regulatory nodes are now clearer. TWEAK/Fn14 signalling has dose-dependent effects on PDLSC proliferation, osteogenesis and their ability to support pro-reparative macrophage activity (Xiao et al., 2026). Kaempferol has been reported to restore impaired osteogenic differentiation through EphrinB2-dependent PI3K/Akt and p38 signalling (Cao et al., 2025). PTEN-associated proteomic changes and non-coding RNAs add further regulation, influencing how PDLSCs commit to specific lineages (Phothichailert et al., 2026; Zhai et al., 2024).

Cellular senescence is a related but distinct barrier to regeneration. Multi-omics studies have linked TGF-beta signalling to senescence programmes in periodontal stem cells (Li B. et al., 2025). In diabetic periodontitis, spermidine counteracted PDLSC senescence by promoting mitophagy (Zhou et al., 2025). Cordycepin-loaded adhesive hydrogel microspheres similarly reduced inflammation-induced premature senescence through NRF2, while improving cell migration, osteogenesis, ligament-forming potential and bone repair (Wang et al., 2025). Together, these data suggest that restoring PDLSC function will require more than growth-factor delivery. Effective regeneration also requires modulation of senescence, mitochondrial dysfunction, oxidative stress and persistent inflammatory priming.

### 2.2 Macrophage-stromal crosstalk beyond M1/M2 polarization

Macrophages have a major role in deciding whether periodontal lesions move towards tissue destruction or repair. Bacterial challenge may initiate periodontitis, but much of the subsequent damage comes from a host immune response that becomes excessive, persistent or poorly resolved. In this setting, macrophages contribute to pathogen clearance, antigen presentation, cytokine release, matrix degradation and tissue breakdown, but they are also needed for later repair (Yamaguchi, 2026; Peng et al., 2024; González-Quintanilla et al., 2026). The familiar M1/M2 framework remains useful as a broad shorthand. It is too simple, however, to describe the range of macrophage states that shape periodontal regeneration. A reparative macrophage response must be timed, locally restricted and compatible with antimicrobial defence, osteoclast control, angiogenesis and matrix remodelling.

The osteoimmune link is central. RANKL-driven osteoclastogenesis connects immune activation with alveolar bone loss (Tsukasaki, 2021). Single-cell analysis shows that periodontitis and initial therapy reshape fibroblast, mesenchymal, monocytic, endothelial, T-cell and B-cell compartments (Chen et al., 2022). Some macrophage subsets may support repair. Cluster of differentiation 301 b (CD301 b)-positive macrophages, for example, have been linked to enhanced bone regeneration in periodontitis treatment (Wang C. et al., 2023).

Macrophage states can also be redirected. Induced pluripotent stem cell-derived mesenchymal stem cells (MSCs) reduced M1 polarization and alveolar bone loss, suggesting that stem-cell therapies may act partly through immune modulation rather than engraftment (Chen et al., 2025). MSC-derived apoptotic bodies restrained osteoclast differentiation and bone destruction (Li et al., 2023). Tumor necrosis factor-alpha (TNF-alpha)-treated gingiva-derived MSC exosomes enhanced M2 polarization and reduced periodontal bone loss (Nakao et al., 2021). Melatonin-engineered M2 macrophage exosomes further promoted immune reprogramming and relieved endoplasmic reticulum stress (Cui et al., 2023). Together, these findings show that stromal-macrophage communication can be therapeutically designed.

The role of macrophages is not limited to inflammatory control; it is also linked to repair programs that resemble developmental processes. When the immune microenvironment shaped by macrophages is properly adjusted, PDL cells may enter a “re-development state” (Liu G. et al., 2024). Within periodontal lesions, immunomodulation is not simply anti-inflammatory; it helps steer the signals needed for tissue reconstruction. For translation, macrophage reprogramming should therefore be judged by downstream stromal outcomes, including progenitor condensation, collagen alignment, cementogenic differentiation and functional PDL insertion, not only by TNF-alpha, interleukin-1 beta (IL-1 beta) or interleukin-10 (IL-10) changes.

### 2.3 Endothelial-driven angiogenesis and progenitor condensation

Periodontal regeneration requires vascularization. New bone, PDL and cementum formation depend on oxygen supply, nutrient exchange, immune-cell trafficking and endothelial-derived signals. Single-cell transcriptomic work identified platelet-derived growth factor receptor alpha (PDGFRA)-positive progenitors that coordinate angiogenesis with periodontal tissue regeneration (Liu J. et al., 2025). This gives cellular specificity to the idea that progenitor condensation and vascular organization are core elements of regenerative patterning. It also explains why osteogenic-only strategies may be insufficient.

Several biomaterials now target osteogenesis and angiogenesis together. A dimethyloxalylglycine/nanosilicate fibrous structure was designed as a dual osteogenic-angiogenic platform for functional periodontal regeneration (Shang et al., 2021). A metal-organic framework (MOF)-modified injectable hydrogel released zinc ions and quercetin, combining antibacterial, hemostatic, immunomodulatory, pro-osteogenic, pro-angiogenic and cell-recruiting effects (Yang S. et al., 2024). This study also linked impaired PDLSC osteoangiogenesis in periodontitis to reduced energy metabolism, oxidative stress and altered autophagy (Yang S. et al., 2024). In diabetic periodontal bone healing, a polyphenol-mediated redox-active hydrogel with hydrogen sulfide release and conductive components promoted MSC recruitment, angiogenesis, osteogenesis and bioelectric communication (Fang et al., 2024).

Angiogenesis should therefore be treated as part of a multi-lineage repair program. A material may first need antibacterial and hemostatic activity, then support immune resolution, vascular ingrowth and matrix maturation. Poorly timed angiogenic cues may be detrimental, as premature stimulation within an inflamed lesion can promote unstable vessel formation, while delayed vascularization may constrain osteogenic condensation. Time-resolved single-cell and spatial analyses are therefore needed to delineate the precise temporal sequence of these events.

### 2.4 Extracellular vesicles, apoptotic bodies, and mitochondrial signalling

Extracellular vesicles (EVs), exosomes and apoptotic bodies are key mediators of cell-free periodontal regeneration. They deliver biological signals while avoiding some risks of living-cell transplantation. MSC-derived exosomes and conditioned media contain growth factors, cytokines, chemokines, enzymes, microRNAs (miRNAs) and other cargoes that can regulate inflammation, osteogenesis, angiogenesis and repair (Ahmad et al., 2025; Wang et al., 2024; Lin et al., 2022; Lin et al., 2021). PDLSC-derived exosomes are especially relevant. In inflammatory PDLSCs, they restored osteogenic differentiation by suppressing excessive canonical Wnt signalling, and they promoted alveolar bone repair in rat periodontitis defects (Lei et al., 2022).

Yet EVs are not inherently regenerative. Engineered EVs may restore periodontal homeostasis through antibacterial, antioxidant, immunomodulatory and reparative actions (Shi et al., 2026). Disease-derived vesicles can have the opposite effect. Liver-derived exosomes aggravated alveolar bone loss in type 2 diabetes through fatty acid synthase (Fasn) transfer (Liu J. et al., 2024). Macrophage-derived mitochondria-rich EVs transferred damaged mitochondria to bone marrow mesenchymal stem cells (BMSCs), disrupted mitochondrial dynamics and impaired osteogenesis (Yan et al., 2025). Bacterial EVs may also connect periodontal inflammation with systemic disease through the oral-gut axis (He et al., 2026). Thus, vesicle function is shaped by the source cell type, priming status, molecular cargo, recipient cell population, and disease-specific microenvironment.

Apoptotic vesicles follow the same logic. MSC-derived apoptotic bodies reduced osteoclast differentiation and alveolar bone destruction (Li et al., 2023). Pyruvate kinase M2 (PKM2)-positive apoptotic vesicle-mediated senolytics improved chronic periodontitis by targeting senescent-cell accumulation and systemic inflammation (Hao et al., 2025). Vesicle-based therapy may therefore reshape periodontal communication, but clinical use still requires standardized manufacturing, molecular cargo profiling, biodistribution testing, safety assessment and potency assays.

## 3 Extracellular matrix remodelling and biomaterial control

This chapter classifies biomaterial strategies by biological function rather than by material class. We focus on how these systems sense the diseased extracellular matrix, retain and release therapeutic cues in wet periodontal defects, convert mechanical or electrical inputs into cell-instructive signals, and rebuild the spatial architecture of the cementum-PDL-bone complex.

The material examples are therefore grouped into four functional categories: disease-responsive matrix sensing, local retention with sequential delivery, electro-mechanical cue conversion, and compartmentalized architecture for functional integration. Each category is evaluated by the biological process it is designed to control, rather than by whether the platform is labelled as a hydrogel, MOF, nanozyme, vesicle system or piezoelectric scaffold.

### 3.1 The extracellular matrix as a dynamic regulator

The periodontal ECM is more than a load-bearing scaffold. It positions cells, transmits mechanical cues, binds growth factors and releases bioactive fragments during degradation, allowing it to shape both inflammation and repair. This regulatory function is particularly evident in the PDL, where tissues are repeatedly subjected to occlusal forces. PDL cells can translate these mechanical inputs into biochemical responses (Zheng et al., 2026). Extracellular adenosine triphosphate (ATP) is another relevant signal, as it modulates periodontal ligament cell behaviour and may contribute to inflammatory signalling (Kyawsoewin et al., 2023). Evidence from magnetic and laser stimulation experiments further indicates that physical stimulation can reshape PDLSC activity (Ponnaiyan et al., 2024; Peluso et al., 2021).

Matrix remodelling can, however, become part of the disease process. During periodontitis, matrix metalloproteinases break down collagen and other ECM constituents, whereas ROS and bacterial products interfere with normal cell-matrix communication. These pathological cues can be leveraged in biomaterial design, where systems responsive to matrix metalloproteinases (MMPs), reactive oxygen species (ROS), pH, or temperature use the disease microenvironment as an endogenous trigger for controlled release. For example, a metal-phenolic nanozyme platform exploited inflammatory charge and MMP activity to enhance local retention and release, while providing antibacterial, antibiofilm, ROS-scavenging, macrophage-polarizing and osteogenic functions (Xu Y. et al., 2023). A dynamic hydrogel-MOF system followed a similar design principle, responding to ROS and pH to release magnesium ions and gallic acid, thereby reducing inflammatory mediators and supporting bone regeneration (Luo et al., 2024). ECM-inspired materials are therefore better described as disease-responsive sensors than as inert scaffolds.

### 3.2 Local retention and sequential delivery in irregular defects

Under this principle, hydrogels and injectable matrices are best viewed as retention and timed-release systems, rather than as a single material class. Their mechanistic value depends on whether they sustain therapeutic concentrations in wet defects, protect labile cues, align release with inflammatory or redox states, and degrade in step with matrix repair.

Hydrogels are widely used in periodontal biomaterial research because they fit irregular defects, retain drugs at the lesion site, protect unstable bioactive molecules and partly mimic the hydrated ECM. Recent reviews describe designs based on natural or synthetic polymer networks, injectable gels, adhesive systems and composite scaffolds (Santos et al., 2023; Pan et al., 2025; Sun et al., 2024). In many current studies, these materials are being developed as active therapeutic platforms rather than simple delivery vehicles. They integrate antibacterial activity, redox control, immunomodulation, osteogenesis, angiogenesis and controlled degradation.

Several platforms illustrate this shift. A zeolitic imidazolate framework-8/gelatin methacryloyl (ZIF-8/GelMA) injectable photopolymerizable hydrogel released zinc ions, inhibited Porphyromonas gingivalis, increased osteogenic gene expression and promoted alveolar bone regeneration *in vivo* (Liu et al., 2022). A five-in-one MOF-modified hydrogel combined thermosensitive adhesion, washout resistance, hemostasis and zinc/quercetin release to support antimicrobial, immunomodulatory, osteogenic, angiogenic and cell-recruiting effects (Yang S. et al., 2024). A thermosensitive nanocomposite hydrogel carrying doxycycline-loaded, folic acid-modified mesoporous bioactive glass nanoparticles promoted M2 macrophage polarization, PDLSC differentiation, bone regeneration and inflammatory niche remodelling (Yu et al., 2026).

Hydrogels are particularly relevant in diabetic periodontitis, where oxidative stress, impaired MSC function and immune dysregulation restrict repair. Zinc-vanadium-silicon-calcium (Zn-V-Si-Ca) glass hydrogel microneedles provided antibacterial and antioxidant effects, suppressed Janus kinase-signal transducer and activator of transcription (JAK-STAT) and NF-kappaB signalling, and improved bone regeneration (Li L. et al., 2025). A redox-active, hydrogen sulfide-releasing conductive hydrogel restored antioxidant balance, immune regulation, MSC recruitment, angiogenesis, osteogenesis and endogenous bioelectricity (Fang et al., 2024). Quercetin-loaded bioglass hydrogels and carotene liposome-responsive hydrogels further support metabolic and redox control as design priorities in systemic-risk settings (Huang et al., 2025; Zhu et al., 2024).

### 3.3 Electro-mechanical cue conversion and mechanotransduction

For bioelectric and piezoelectric systems, the key mechanistic question is how physical loading is converted into cell-instructive signals. In periodontal defects, these platforms are most relevant when they link occlusal mechanics to ATP production, macrophage state, PDLSC metabolism and matrix organization.

The PDL is mechanically active, and new biomaterials are beginning to exploit this feature. Piezoelectric hydrogels convert local mechanical stress into electrical cues. One such hydrogel rescued the osteogenic capacity of inflammatory PDLSCs by improving energy metabolism and ATP synthesis, while also shifting macrophages toward an anti-inflammatory and pro-regenerative phenotype (Liu X. et al., 2024). Injectable piezoelectric hydrogels provide a related strategy for periodontal disease treatment (Roldan et al., 2023). A biodegradable piezoelectric Janus membrane further combined antibacterial and osteogenic functions in periodontal applications (Li K. et al., 2025). Beyond piezoelectricity, magnetoelectric coupling stimulation has been used to reprogram macrophages during infected periodontal tissue regeneration (Liu et al., 2026).

These studies connect mechanics, bioelectricity, metabolism and immunity. They suggest that regeneration may benefit from cues closer to physiological PDL loading, rather than relying only on static osteoinductive signals. Still, the field lacks clear parameters for dose, duration, frequency and spatial distribution of bioelectric stimulation. Suboptimal stimulation (in intensity or timing) may exacerbate local inflammation or interfere with normal matrix organisation. Future models should therefore be capable of measuring mechanical loading in addition to cellular metabolism and immune status instead of considering them as alternative outcomes.

The mechanically active PDL forms a distinct regenerative microenvironment. During mastication and tooth movement, PDL cells are exposed to repeated tensile, compressive and shear forces. These cues can regulate cytoskeletal organization, ATP release, Wnt/beta-catenin activity, matrix remodelling and lineage commitment. Within a physiological range, mechanical loading may support fibre alignment, cementum-PDL-bone integration and functional maturation. By contrast, excessive or poorly timed loading may amplify inflammation, disrupt collagen organization and destabilize newly formed tissue. Periodontal regenerative strategies should therefore treat mechanical loading as an active design variable, not a background condition. Materials with appropriate stiffness, degradation kinetics, fibre-guiding architecture and mechanoresponsive or piezoelectric properties may better match the mechanosensitive PDL niche. Evaluation should also extend beyond bone fill to include force-related endpoints, including PDL width, collagen fibre orientation, Sharpey-like fibre insertion and mechanical integration (Connizzo et al., 2021; Kyawsoewin et al., 2023; Ono et al., 2025; Zheng et al., 2026).

### 3.4 Compartmentalized architecture for functional integration

The final principle is spatial compartmentalization. Periodontal repair must regenerate mineralized and fibrous tissues in register, so scaffold architecture should provide regional control over stiffness, porosity, degradation, mineral content and fibre guidance, rather than deliver a uniform cue throughout the defect.

The periodontium has an architectural pattern of space, making it difficult to reconstruct it with one uniform substance completely. Based on this, a growing number of more recent reviews and investigations of scaffolds have involved the development of multicompartmental or bilayered systems in which bone, PDL and cementum regeneration is coordinated (Chen et al., 2024; Deng et al., 2022; Yamada et al., 2022; Chen and Huang, 2022; Yao et al., 2022). One of those is a hierarchical bilayered scaffold for the regeneration of periodontal complex (Qin et al., 2025). These constructs can have various specific zone values tailored for porosity, degradation rate, stiffness, mineral content and bioactive signalling so catering to the various tissue interfaces in a more ordered fashion (Yao et al., 2022).

This design logic is based in periodontal biology. In alveolar bone repair, there is a need for mineralized matrix to form and for osteogenic stimulation. The PDL, in contrast, requires aligned collagen fibres, a maintained ligament space, vascular ingrowth and sufficient mechanical compliance. Cementum regeneration further relies on cementoblast-like differentiation and the correct insertion of Sharpey-like fibres. For this reason, a scaffold that mainly promotes bone formation may still be inadequate if it cannot re-establish functional periodontal attachment.

A major weakness in many preclinical studies is endpoint selection. Bone volume is easy to quantify, but micro-computed tomography (micro-CT) bone fill does not prove functional regeneration if cementum and oriented PDL fibres are missing. Future work should routinely assess cementum apposition, fibre insertion, PDL width, vascular organization, innervation when relevant and mechanical integration. The Wnt/beta-catenin study of cementum apposition is valuable because it links regeneration to *in vivo* cementum-PDL-bone reconstruction, not bone repair alone (Ono et al., 2025). Table 2 synthesizes how major biomaterial strategies control these niche variables and where their translational risks differ.

**TABLE 2:** Mechanistic classification of biomaterial strategies for periodontal microenvironment control.

Strategy	Main mechanism	Best-fit regenerative task	Key limitation to control
Adhesive/injectable hydrogels	Wet retention, defect filling and sustained local delivery	Irregular periodontal defects needing local depot formation	Burst release, washout and weak mechanical support
Stimuli-responsive matrices	MMP, ROS, pH or temperature-triggered release	Inflamed lesions requiring disease-matched timing	Unpredictable release under heterogeneous inflammation
Ion/MOF systems	Zn, ca, si, V or oxygen release with antibacterial/osteogenic cues	Early microbial control plus stem-cell rescue	Ion dose, persistence and off-target cytotoxicity
Nanozyme/redox platforms	ROS buffering and NRF2/NF-kappab pathway tuning	Oxidative and immune-dysregulated defects	Excess antioxidant effect may weaken host defence
Antibiotic/peptide carriers	Local antimicrobial pressure with reduced systemic exposure	High bacterial burden or early post-surgical contamination risk	Resistance pressure and short therapeutic window
Piezoelectric/bioelectric matrices	Mechanical loading converted into electrical/metabolic cues	PDL-like mechanotransduction and energy rescue	Dose, frequency and long-term stimulation remain unclear
Multicompartment scaffolds	Spatial separation of bone, PDL and cementum cues	Architectural reconstruction of attachment apparatus	Manufacturing complexity and alignment reproducibility
EV/composite systems	Paracrine cargo combined with scaffold retention	Cell-free immunomodulatory and osteogenic signalling	Cargo heterogeneity, potency assays and storage stability

## 4 Immunomodulation from inflammation suppression to resolution engineering

### 4.1 Osteoimmunology and RANKL-Driven bone loss

Periodontitis is increasingly recognized as an osteoimmune disorder characterized by dysregulated interactions between inflammatory and bone-remodelling pathways. Although bacterial dysbiosis triggers the initial inflammatory response, the subsequent loss of alveolar bone is driven mainly by immune-mediated osteoclast activation and inadequate tissue repair. RANKL links immune activation to osteoclastogenesis (Tsukasaki, 2021), which leaves only a narrow therapeutic window. Osteoclast activity must be restrained, but host defence and normal bone remodelling still need to function. Excessive suppression could weaken infection control, whereas insufficient suppression allows destructive bone resorption to continue.

There is a window for immunomodulatory nanotherapeutics and biomaterials to operate. A number of nanotherapeutic approaches have the potential to decrease inflammation and promote periodontal regeneration (Xu T. et al., 2023). Immune-directed, therapies more widely are also interesting although clinical significance is will depend on cellular/molecular specificity and safety as well as timing. This is because numerous recently developed material systems are conceived in conjunction with reprogramming the immune system, in addition to being antibacterial. Shrinking the inflammation with out killing off the microbes is probably going to provide only a short term benefit and may increase the risk of the lesion from recurring.

Accordingly, immune regulation in periodontal regeneration should not be framed solely around macrophage polarization. Neutrophils dominate the early antimicrobial phase, but excessive neutrophil activity can amplify ROS release, protease-mediated matrix damage and cytokine signalling. Regenerative materials should therefore limit neutrophil-driven tissue injury while preserving bacterial clearance. Adaptive immune cells provide a further layer of osteoimmune control. T-cell subsets, particularly the Th17/Treg balance, shape whether inflammation persists or resolves, whereas B cells and plasma cells can support local antibody responses and supply RANKL for osteoclastogenesis. The T/B-cell-RANKL axis, already summarized in Table 1, links immune-cell composition to the RANKL/OPG balance and bone turnover. Thus, balanced immunomodulatory design should assess macrophage phenotype together with neutrophil activity, T-cell polarization, B-cell and plasma-cell responses, RANKL/OPG balance and osteoclast activity. These readouts are needed to coordinate antibacterial defence, inflammatory resolution and bone regeneration (Tsukasaki, 2021; Chen et al., 2022).

### 4.2 Macrophage reprogramming as a biomaterial design principle

The macrophage-directed immunotherapy by biomaterial approach has become a common strategy in periodontal regeneration (Peng et al., 2024). The goal is to promote a transition of macrophage activity from the tissue damaging inflammation to states which will allow resolution and repair. However, this cannot simply be translated into a push towards the stable M2 phenotype. The healing process of the periodontium is compartmentalised. Early bacterial clearance, removal of debris, inflammatory resolution, production of matrix, angiogenesis and subsequent tissue remodelling are required. Early M2 polarization, however, can lead to reduced antimicrobial responses while sustained M1 polarization can impact stem cell differentiation and vessel maturation.

Macrophage membrane-camouflaged nanodecoys are an example of this ‘staged’ strategy. The coating accounted for enhanced targeting and retention to the bacteria, the system also was associated with the improvement of oxygen generation under hypoxia, NRF2 activation, oxidative-stress control, and macrophage phenotype reprogramming (Lin et al., 2025). Here, components derived from macrophages provide more than a guide for delivery. They supply an immune-regulatory interface. The general value that comes from this is that antibacterial, antioxidant and immunomodulatory activities should work synergistically rather than being therapeutic activities done separately within the periodontal pocket.

Other material platforms work in a identical way. Metal phenolics nanozymes show the ability to regulate macrophage polarization by pathways connected to NRF2/NF-kappaB (Xu Y. et al., 2023). Thermosensitive hydrogels with doxycycline induces phenotype control in macrophages and contributes to PDLSC differentiation (Yu et al., 2026). In diabetic periodontitis, bioactive glass hydrogel microneedles bring down inflammatory signalling (Li L. et al., 2025). There is another way; given that exosomes produced by M2 macrophage engineered to express melatonin and home to inflammatory sites have the ability to alleviate endoplasmic reticulum stress (Cui et al., 2023). While these methods vary in terms of the materials they use and the cargo they deliver, they both have therapeutic goals: to hijack ongoing immune activation and push it toward positive immune resolution.

### 4.3 Redox homeostasis and the NRF2/NF-kappab axis

Oxidative stress is a common driver of periodontal regenerative failure. Excess ROS damages cells and matrix, activates inflammatory pathways, impairs mitochondria, promotes senescence and suppresses osteogenesis. Yet redox signals also participate in host defence and repair. Antioxidant therapy should therefore be local, timed and balanced, not indiscriminate.

NRF2 is a recurrent protective node. Adhesive hydrogel microspheres activated NRF2-related programs, reducing deoxyribonucleic acid (DNA) injury and premature senescence in PDLSCs (Wang et al., 2025). Metal-phenolic nanozymes and macrophage membrane nanodecoys also used NRF2/NF-kappaB-linked mechanisms to limit oxidative stress and inflammatory polarization (Lin et al., 2025; Xu Y. et al., 2023). Redox control is especially important in diabetes, where hyperglycemia intensifies oxidative injury, immune imbalance and stem-cell dysfunction (Zhou et al., 2025; Li L. et al., 2025; Fang et al., 2024).

Together, these studies suggest that regenerative materials should be tested for cell-specific redox repair. Relevant readouts should include PDLSC survival and osteogenesis, macrophage polarization, endothelial function and osteoblast-lineage activity. The goal is not simply to remove ROS, but to restore redox homeostasis within the periodontal regenerative niche.

### 4.4 Senescence and systemic inflammatory coupling

Senescence links inflammation, oxidative stress, mitochondrial dysfunction and impaired repair. In chronic periodontitis, local and systemic inflammation may promote apoptotic resistance and senescent-cell accumulation (Hao et al., 2025). PKM2-positive apoptotic vesicle-mediated senolytics improved chronic periodontitis in experimental models, suggesting that senescent burden is therapeutically targetable (Hao et al., 2025). Stromal senescence is also therapeutically modifiable, as TGF-beta signalling promotes senescence in periodontal stem cells, whereas activation of mitophagy can attenuate PDLSC senescence in diabetic periodontitis (Li B. et al., 2025; Zhou et al., 2025).

Senescence should therefore be viewed as more than a marker of tissue aging. Through senescence-associated secretory phenotypes, impaired differentiation, altered ECM remodelling and defective clearance, senescent cells may help maintain a pro-inflammatory and anti-regenerative niche. Therapies that reduce senescent-cell burden or restore mitochondrial quality control could strengthen conventional periodontal regeneration.

## 5 Metabolic and mitochondrial control of regeneration

Mitochondria are now central to PDLSC biology. Mitochondrial ROS, dynamics, mitophagy, glucose metabolism and tricarboxylic-acid-cycle metabolites regulate PDLSC osteogenesis, senescence, inflammation, apoptosis and regenerative capacity (Zhao et al., 2026). This framework connects findings that otherwise appear separate. Inflammatory cytokines, diabetes, hypoxia, oxidative stress and bioelectric stimulation all alter mitochondrial function. In turn, mitochondrial state determines whether PDLSCs can proliferate, migrate, differentiate and communicate with immune cells.

Mitophagy is especially important in diabetic periodontitis. Spermidine reversed PDLSC senescence through mitophagy and alleviated diabetic periodontal damage (Zhou et al., 2025). By contrast, macrophage-derived mitochondria-rich EVs transferred damaged mitochondria to BMSCs, disrupted mitochondrial dynamics and impaired osteogenesis (Yan et al., 2025). Piezoelectric stimulation increased ATP synthesis and energy metabolism in inflammatory PDLSCs (Liu X. et al., 2024). These findings support the principle that regenerative niches should restore metabolic competence rather than merely suppress inflammation.

**FIGURE 2:**
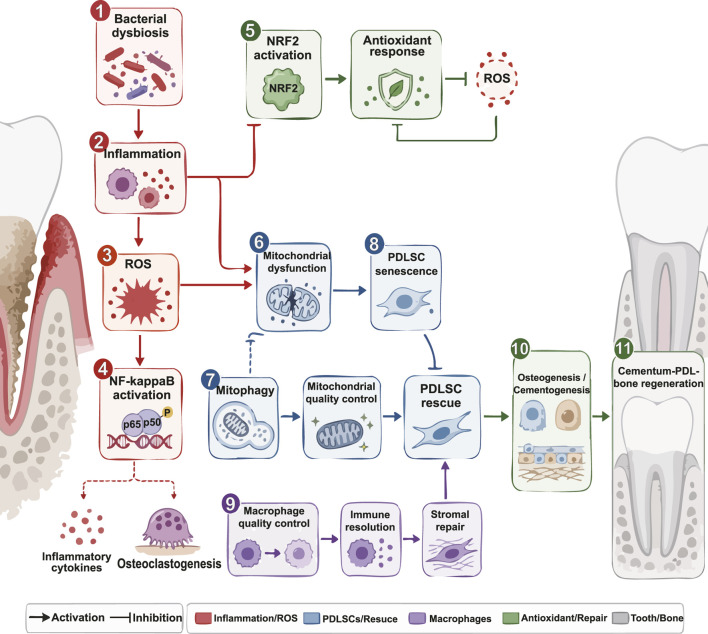
Redox, mitochondrial and immune-resolution control of periodontal regeneration. Bacterial dysbiosis and inflammation increase ROS production and NF-kappaB signalling. This inflammatory-redox axis promotes cytokine release, osteoclastogenesis, mitochondrial dysfunction and PDLSC senescence. In the reparative direction, NRF2 activation, antioxidant responses, mitophagy-based mitochondrial quality control and macrophage reprogramming support immune resolution, PDLSC rescue, osteogenesis/cementogenesis and cementum-PDL-bone regeneration.

Hydrogel studies support the same view. A five-in-one MOF-modified hydrogel reversed periodontitis-associated suppression of PDLSC energy metabolism and corrected oxidative-stress and autophagy abnormalities (Yang S. et al., 2024). Bioelectric and conductive hydrogels further use endogenous electrical and metabolic processes rather than acting only as soluble-factor depots (Liu X. et al., 2024; Fang et al., 2024). Future work should define metabolic biomarkers of regenerative competence, including mitochondrial membrane potential, mitophagy flux, ATP production, glycolytic-oxidative balance and redox-buffering capacity. This repair logic is outlined in Figure 2. Inflammatory ROS and NF-kappaB activation are linked to mitochondrial dysfunction and PDLSC senescence. By contrast, NRF2 activation, mitophagy and macrophage reprogramming shift the niche toward stromal rescue and cementum-PDL-bone regeneration.

## 6 Toward integrated therapeutic design

The reviewed literature supports an integrated design paradigm for periodontal regeneration. The initial priority is to mitigate dysbiosis. Antibacterial ions, antimicrobial peptides, doxycycline, ZIF-8, metal-organic frameworks (MOFs), nanozymes, and macrophage membrane-based systems have all been developed to reduce the local microbial burden (Lin et al., 2025; Yang S. et al., 2024; Yu et al., 2026; Xu Y. et al., 2023; Liu et al., 2022). The second step is immune resolution, not broad immunosuppression. This requires macrophage reprogramming, ROS control, NRF2 activation and osteoclast restraint (Lin et al., 2025; Peng et al., 2024; Wang C. et al., 2023; Wang et al., 2025; Xu Y. et al., 2023). In diabetic or high-oxidative-stress defects, redox and inflammatory control become even more important (Li L. et al., 2025; Cui et al., 2023).

The third step is rescue of resident regenerative cells. PDLSCs and progenitors require mitochondrial recovery, reduced senescence, osteogenic or cementogenic cues and a supportive ECM (Li B. et al., 2025; Zhou et al., 2025; Cao et al., 2025; Wang et al., 2025; Liu G. et al., 2024). PDGFRA-positive progenitors and Wnt/beta-catenin-mediated cementum apposition further show that developmental pathways can coordinate regeneration (Liu J. et al., 2025; Ono et al., 2025). The fourth step is architectural maturation. Angiogenesis, collagen organization, cementum formation and compartment-specific scaffold design should be treated as essential outcomes, not secondary endpoints (Shang et al., 2021; Fang et al., 2024; Qin et al., 2025; Yao et al., 2022).

These requirements make multifunctional materials attractive, but stacking of functionalities can also provide one with a more complex problem in trying to control system. A stronger strategy of design is to build material responsiveness along the biological pathway of periodontal healing, which involves periodontal pocket retention, early antimicrobial and hemostatic actions, responsiveness to disease signals, immune resolution, recruitment of stem cells, inducing osteogenesis, inducing angiogenesis, inducing cementogenesis, and matrix maturation. For each stage there should be specified biomarkers and an expected time frame of the stage. Other cargoes without such temporal or molecular stratification might create the variability as opposed to improving regenerative outcomes.

Some issues, such as poor cell survival and immunogenicity (Wang et al., 2024; Lin et al., 2022) in direct MSC transplantation may be avoided in cell-free approaches, particularly in EVs and conditioned media. But, their benefits rely upon the source and condition of the donor cells. Diseased tissues or inflammatory macrophages can provide vesicles with cargo that stimulates disease processes (He et al., 2026; Yan et al., 2025; Liu J. et al., 2024). Targeting or function may be improved with Preconditioning and Engineering; translation will need standardized ways of isolating, cargo profiling, Potency assays, storage conditions, Dosing rules and Safety tests (Shi et al., 2026; Lin et al., 2021).

Developmental engineering puts the science of engineering into a more specific end. The aim is not merely to lay down mineralized tissue, but to promote a functional periodontal attachment apparatus. One connection between immune control and developmental repair is the macrophage regulation which takes PDL cells into a re-developmental state (Liu G. et al., 2024). Therefore, more mature therapies would require such a control of both space and time.

## 7 Broadening the translational evidence base

Adopted clinical modalities should not be forgotten in an integrated regenerative sequence. Enamel matrix derivative continues to enjoy value, as the relationship between it and periodontal effects can be modified by diabetes, demonstrating the influence of host metabolism in response to bioactive matrices (Narita et al., 2021). All platelet concentrates, such as PRP, PRF and CGF, deliver autogenous growth factors in a fibrine matrix. They can best be considered, however, as part of the wider microenvironmental programme than separate regenerative therapies (Mijiritsky et al., 2021). Alloplastic grafts, barrier membranes, nanomaterial scaffolds or the oral-maxillofacial bone substitutes still contribute to space maintenance and to guided tissue regeneration and to structural support (Li et al., 2024; Ashfaq et al., 2024; Chen and Huang, 2022; Varghese et al., 2022).

All of this is subject to a lot of clinical context. Plaque control, defect shape, smoking status, diabetes, tooth mobility and surgical accessibility all affect whether an advanced material can express its intended biological function (Levine et al., 2024). Evidence from guided tissue regeneration combined with microscrew implant anchorage further suggests that periodontal repair, in malocclusion cases, must be coordinated with force control and defect management (Liang et al., 2025). Locally repurposed pharmaceuticals may also serve as useful adjuncts when their antimicrobial, anti-inflammatory or osteogenic actions fit the local defect microenvironment (El-Nablaway et al., 2024).

Recent studies have broadened material design beyond simple hydrogel-based release. Immunomodulatory microneedle patches and environmentally responsive hydrogels illustrate how staged release can be adjusted to the inflammatory state of periodontal tissue (Zhang X. et al., 2022; Qiu et al., 2026). Sericin-based microspheres, bone-healing-cascade delivery systems and mitochondrial-targeted injectable hydrogels coordinate flora regulation, oxidative stress, macrophage behavior and stem-cell fitness (Zhou et al., 2026; Su et al., 2026; Wang H. et al., 2023). Glycopeptide hydrogels and nano-hydroxyapatite/chitosan bioaerogels combine osteoclast restraint, osteogenesis and mineralized matrix formation with scaffold cues (Zhao et al., 2024; Souto-Lopes et al., 2024). Bio-nano architectures further treat infection control as an engineered function (Du et al., 2026).

Biological cargoes also broaden the model. Dental-pulp-stem-cell exosomes regulate anti-inflammatory and osteogenic responses, while plant-derived exosome-like nanoparticles may act as immune regulators (Qiao et al., 2023; Zhang Z. et al., 2022). Periodontal tissue used after bone morphogenetic protein-2 (*BMP-2*) gene transfer suggests that endogenous matrix cues can be repurposed for hard-tissue repair (Kawai et al., 2022). Pyrophosphate-regulator ablation also points to mineralization control as a target in periodontal regeneration (Nagasaki et al., 2021). These examples extend the stromal-immune-matrix model by adding clinical risk, endogenous matrices, mineral metabolism and precision anti-infective design.

## 8 Temporal logic of periodontal niche reprogramming

The interventions discussed in this review can be read as a staged model of periodontal regeneration rather than as isolated therapeutic options. At the beginning of healing, periodontal defects are not clean wounds. Periodontal defects are chemically and mechanically hostile sites. They contain bacterial products, necrotic debris, ROS, MMPs, inflammatory cytokines and hypoxic regions. Local retention is the first that lacks of practicality under these conditions. Materials should remain in the wet, irregular and mobile areas of the periodontal pockets despite the presence of saliva, crevicular fluid, bleeding and mechanical disturbance. This is solved by application of thermosensitive adhesive hydrogels that gel *in situ*, antibacterial nanocomposite hydrogels that afford additional immunomodulatory and regenerative abilities, and microneedles that can be applied through the mucosal barrier and establish local depots because of their short persistence (Yang S. et al., 2024; Yu et al., 2026; Li L. et al., 2025). Even the most advanced treatment may be more like a flashlight beam if it does not retain.

Once there is a residence in the body, it is no longer a question of achieving complete sterility but reducing microbial and oxidative stress. In the case of ZIF-8/GelMA and zinc-releasing MOF hydrogels, they are able to reduce the amount of bacteria existing in the system while maintaining repair-friendly conditions (Yang S. et al., 2024; Liu et al., 2022). Antimicrobial activity can be imparted by doxycycline-loaded platforms, which can also affect the behaviour of macrophages (Yu et al., 2026). There are other systems that work in more interdependent ways. Macrophage membrane nanodecoys were integrated with bacterial-targeting (here via the targeting bacterial peptide), hypoxia-responsive oxygen production and presentation of antimicrobial peptides and the related NRF2-dependent immunomodulation. Antibacterial property, ROS scavenging and osteogenic support are combined into metal-phenolic nanozymes (Xu Y. et al., 2023). These strategies all work to dampen the vicious circle of hyperactivation by dysbiosis.

After the microbial and redox pressure is lowered immune regulation is crucial. Macrophage immunotherapy via biomaterial is valuable strategic insights for this stage (Peng et al., 2024; Yang et al., 2021). The target is inflammatory resolution, rather than immune suppression. Macrophage immunotherapy via biomaterials is a helpful platform for this step (Peng et al., 2024). Doxycycline hydrogels, metal-phenolic nanozymes, NRF2-protective hydrogel microspheres and melatonin-engineered M2 macrophage exosomes all decrease the inflammatory damage and enhance repair (Yu et al., 2026; Wang et al., 2025; Xu Y. et al., 2023; Cui et al., 2023). This distinction matters. Uncontrolled inflammation can lead to ongoing osteoclastogenesis and damage to the stroma while too much suppression can cause the infection control properties to falter.

Regeneration then would rely on the ability of the stromal compartment to heal. During periodontitis, PDLSCs and PDL fibroblasts are frequently found to have senescence, metabolic dysfunction and decreased osteogenic and/or cementogenic potential (Huang et al., 2024; Jin et al., 2022). The mechanism of stromal rescue has been linked to redox control, mitochondrial quality and lineage differentiation, such as in the case of TGF beta-driven senescence (Zhai et al., 2024; Wang et al., 2025; Lei et al., 2022), non-coding RNAs (Cao et al., 2025) and the exosomal effects of Wnt (Li B. et al., 2025; Cao et al., 2025). Another clue in recruitment of PDGFRA-positive progenitors is that progenitor activation should also be timed to angiogenesis and not considered a distinct event (Liu J. et al., 2025). Hence, bone fill alone is not a complete end-point. True regeneration involves cementum formation, infiltration of PDL fibres into cementum and bone, and matrix maturation with the stimulation of functional loads. These latter structural events seem to be supported by Wnt/beta-catenin signalling and ATP mediated mechanotransduction (Zheng et al., 2026; Ono et al., 2025; Kyawsoewin et al., 2023)).

Piezoelectric, conductive and Janus membrane systems are among the newer class of bioelectric materials, which enable a transformation from mechanical or redox cues into electrical or metabolic signals (Liu X. et al., 2024; Fang et al., 2024; Roldan et al., 2023; Li K. et al., 2025). A single signal is not sufficient for all zones since PDL mechanics, matrix changes, are region dependent (Connizzo et al., 2021). For long-term success, microbial balance, controlled inflammation, vascular function, matrix organization and integration in the mechanical environment will likely need to remain after the end of material degradation and/or drug release. Because of this, the early bone gain could overestimate the regeneration when the cementum-PDL-bone architecture is weak or negatively immune.

## 9 Disease context and evaluation standards

Clinical severity is an important determinant of the periodontal regenerative microenvironment. In mild periodontitis, the regenerative niche may retain a relatively preserved PDL cell pool and vascular supply. The main requirement is therefore early control of dysbiosis and inflammation before matrix breakdown becomes irreversible. Moderate periodontitis presents a more complex niche, characterized by sustained cytokine signalling, partial stem-cell dysfunction, collagen degradation and osteoclast activation. At this stage, immune resolution must be coordinated with angiogenesis and osteogenic or cementogenic repair.

Severe periodontitis presents a higher biological threshold for regeneration. Advanced lesions are more likely to contain deep microbial reservoirs, extensive ECM destruction, reduced regenerative-cell fitness, impaired vascular organization, increased osteoclast activity and mechanically unstable defects. In this setting, a single osteogenic or anti-inflammatory cue is unlikely to be sufficient. Regenerative design should instead combine stronger infection control, staged immunomodulation, stem-cell rescue, matrix reconstruction, vascular support and mechanical stabilization. Endpoints should also test cementum-PDL-bone integration rather than bone fill alone.

It is doubtful that all periodontal defects will have a single regenerative goal. The prime example is diabetes. Hyperglycaemia elevates the oxidative stress causes a malfunction of the MSC and PDLSC, modifies the behaviour of macrophages and accelerates tissue breakdown. So the current research on diabetic periodontitis is not only focussed on osteoinduction but is focussed on a combination of antioxidant protection, immune regulation and stem-cell rescue (Zhou et al., 2025; Li L. et al., 2025; Fang et al., 2024; Liu J. et al., 2024). Spermidine has been linked to mitophagy during this metabolically altered repair setting (Fang et al., 2024; Li L. et al., 2025; Zhou et al., 2025) as have the Zn-V-Si-Ca glass microneedles and hydrogen sulfide/bioelectric hydrogels. There is an added constraint with senescence. It dampens the response of stem cells and maintains inflammatory secretory programmes through TGF beta signalling pathway by a persistent inflammation and mitochondrial dysfunction (Li B. et al., 2025; Zhou et al., 2025; Hao et al., 2025).

Periodontal lesion may be localized, but their biology is influenced by systemic conditions. Diabetic alveolar bone loss, for instance, was exacerbated by liver-derived exosomes transferring the model protein Fasn (He et al., 2026; Liu J. et al., 2024); and bacterial extracellular vesicles could provide a potential association between periodontal inflammation and the oral-gut axis. Mechanics also matter. PDL behaviour is affected by occlusal loading and orthodontic force, implant proximity and tooth mobility. The PDL cells appear to constantly perceive and react to mechanical stress, as evidenced by mechanosensing and extracellular ATP signalling (Zheng et al., 2026; Kyawsoewin et al., 2023). These bioelectric and piezoelectric materials then can function only if the defect is mechanically stable enough to allow reasonable stimulation (Liu X. et al., 2024; Roldan et al., 2023; Li K. et al., 2025).

These modifiers also require more rigorous testing. Just the lower levels of cytokines and higher micro-CT BV cannot be used to judge regeneration. Antimicrobial, macrophage and general immune states, osteoclast activity and involvement in PDLSC or PDL progenitors, angiogenesis, cementum formation, PDL fibre orientation, and long term PDL stability should be assessed. From these cells, states associated with disease, single-cell studies have already identified disease-associated fibroblast and immune states, states of diverse dental mesenchymal populations and PDGFRA + regenerative progenitors (Yang Y. et al., 2024; Chen et al., 2022; Liu J. et al., 2025). Localization in space to the surfaces of the roots, bone, blood vessels, biomaterial remains, collagen bundles and biofilms is the next step.

It will also be important to determine realistically whether these systems can be manufactured for clinical translation. Multifunctional hydrogels, engineered EVs and macrophage membranes, nanozymes, and piezoelectric materials are attractive, but the more components added, the more variable, regulated and safety issues. Mechanism defined products should be explicit about the product’s activity, whether or not this activity is an essential part of the product and whether a simpler product could achieve the same biological shift. In the case of therapies that involve EVs, the potency assays should include cargo consistency as well as a functional output such as macrophage reprogramming, PDLSC osteogenic rescue, and inflammatory suppression (Shi et al., 2026; Ahmad et al., 2025; Wang et al., 2024; Lei et al., 2022; Nakao et al., 2021). For hydrogels the release should not only be given in terms of material performance but in terms of synergy with biological time windows. This sequence is translated in a useful framework in Table 3, where we pair the phase of fixing the intact tissue with intervention, type, and the minimal evaluation read-out.

**TABLE 3:** Temporal design and evaluation framework for periodontal regeneration.

Phase	Biological objective	Candidate intervention/Readout	Failure signal
Retention/protection	Keep therapy in wet, irregular and enzyme-rich defects	Adhesion, injectability, degradation and residence-time testing	Early washout or material collapse
Microbial control	Reduce dysbiosis without eliminating repair-compatible immunity	CFU/biofilm assays, antimicrobial ions, peptides or antibiotics	Sterile site but delayed healing
Redox-metabolic rescue	Lower damaging ROS and restore mitochondrial fitness	ROS, NRF2, mitophagy, ATP and senescence markers	Persistent oxidative stress or exhausted progenitors
Immune resolution	Shift from destructive inflammation to repair-supportive signalling	Macrophage states, cytokine panels, RANKL/OPG and osteoclast assays	Ongoing osteoclastogenesis and cytokine dominance
Progenitor recruitment	Recover PDLSC/fibroblast function and vascular support	PDLSC markers, angiogenic fronts, PDGFRA + progenitors and proliferation	Poor cell survival or weak lineage commitment
Matrix maturation	Build cementum-PDL-bone architecture under loading	Cementum apposition, sharpey-like fibres, PDL width and micro-CT	Bone fill without organized attachment
Long-term stability	Maintain a relapse-resistant regenerative niche	Functional loading, inflammation recurrence and pocket stability	Recurrent pocket breakdown or inflammatory rebound

## 10 Knowledge gaps and future directions

There are still some obstacles to overcome before the experimental regeneration of periodontal tissue can be used routinely in the clinic. The first is in terms of comparability. Different animal models and defect designs, different bacterial challenge methods, and different measures of outcome significantly limit the ability to compare one strategy with another. Inflammatory and diabetic periodontitis models are needed to be more standardized, particularly to test efficacy against the different disease backgrounds. Evaluation should not only consider micro-CT bone gain. Mechanical function and long-term durability should be present in a regenerated site as well as cementum formation, PDL fibre insertion and width, collagen alignment, vascular organization.

Mechanistic resolution is still limited. Longitudinal single-cell and spatial profiling could track the shift from disease to repair and identify targetable spatial states, such as inflammatory fibroblast niches, macrophage-progenitor contact zones, angiogenic fronts and cementogenic interfaces. Also, a more precise term of the immune-regulation is required. Future studies should not primarily rely on M1/M2 labelling, but rather should define macrophage states based on transcriptomic/functional and metabolic characteristics. Only during the late stages of validation, however, systemic modifiers such as diabetes, ageing, liver dysfunction and chronic inflammation should be considered for material design.

It is as well important to control the product and stratify patients. For EV-based therapies, there is a lack of definition of the state of the source cells, what kinds of cells affect the vesicles, what would the vesicles contain, and whether there would be disease-derived substances as contaminations in the vesicles. Dose-response relationships and safety windows are not well established with bioelectric, piezoelectric, magnetic and mechanically active systems. Clinically, all factors of defect morphology, plaque control, smoking, diabetes, occlusal loading, surgical technique and patient’s adherence affect the results. Advanced materials may also not be able to compensate for uncontrolled clinical conditions, although better diagnostics may help match specific therapies to such inflamed, senescent, diabetic or mechanically compromised defects.

## 11 Conclusion

The stromal, immune, vascular, osteoclastic and matrix derived signals form the periodontal regenerative niche. Although PDLSCs and PDL fibroblasts can serve as the cellular foundation for repair, inflammation, senescence, mitochondrial dysfunction and oxidative stress can decrease the regenerative function of these cells. Macrophages are more context dependent. They can increase tissue damage, but they can also help with resolution, pro-angiogenesis and help repair. The communication of this niche is also influenced by EVs, apoptotic bodies, mitochondrial transfer, ECM remodelling and biomaterials, via cargo delivery, mechanical cues, ion release, redox control, immune polarization and cell recruitment.

Thus, periodontal regeneration should not be thought as a mere process of tissue replacement but rather microenvironmental reprogramming. Higher efficacy has been improved therapies that step by step coordinate the antimicrobial control, the immune resolution, the stem-cell rescue, the angiogenesis and the cementogenesis and matrix organization. The next step is to move beyond broadly multifunctional platforms and develop mechanism-defined, standardized and clinically realistic strategies for rebuilding the cementum-PDL-bone complex.
